# The Effects of Lateral Bounds on Post-Activation Potentiation of Change-of-Direction Speed Measured by the 505 Test in College-Aged Men and Women

**DOI:** 10.3390/sports8050071

**Published:** 2020-05-22

**Authors:** Ashley J. Orjalo, Robert G. Lockie, Katherine Balfany, Samuel J. Callaghan

**Affiliations:** 1 Department of Kinesiology, California State University, Fullerton, CA 92831, USA; ashley.orjalo@csu.fullerton.edu (A.J.O.); rlockie@fullerton.edu (R.G.L.); 2 Department of Physical Medicine and Rehabilitation, University of Colorado, Anschutz Medical Campus, Aurora, CO 80045, USA; katherine.balfany@cuanschutz.edu; 3 Centre for Sports and Exercise Science Research, School of Medical and Health Sciences, Edith Cowan University, Joondalup, WA 6027, Australia

**Keywords:** agility, college-aged, complex training, lateral jump, plyometrics, power training

## Abstract

Forty recreationally-trained individuals completed four testing sessions to determine whether lateral bounds (LB) or weighted lateral bounds enhanced change-of-direction (COD) speed measured by the 505 COD speed test. Session 1 included vertical jump and lateral bound (LB) testing to measure power. Sessions 2–4 involved three randomized conditioning activities (CA): 3 × 5 LB; 3 × 5 weighted LB (10% body mass provided by a weighted vest); and a control condition (4-min rest). The 505 COD speed test was performed 5- and 2.5-min pre-CA, and ~15 s, 4, 8, 12, and 16 min post-CA. A 3 × 6 repeated measures analysis of variance (ANOVA) calculated performance changes across time points post-CA. A 3 × 2 repeated measures ANOVA analyzed best potentiated performance. Smallest worthwhile change (SWC) measured within-subject 505 COD speed test performance. Partial correlations controlling for sex calculated relationships between the vertical jump, LB, and percent potentiation. There were no differences (*p* = 0.919) in 505 time relative to baseline for any CA, nor was the SWC exceeded. The best potentiated 505 time was faster (*p* < 0.001) than baseline for all CA, with no between-CA differences. There were no significant (*p* = 0.056–0.993) correlations between power and potentiation. LB and weighted LB did not potentiate the 505 COD speed test, although performance was not hindered.

## 1. Introduction

Change-of-direction (COD) speed is a vital quality for many athletes. Rapid and decisive COD movements are performed throughout match-play and are often critical to individual and team success for many field- and court-based sports (e.g., a line break in rugby, a fast break in basketball, a score or shift in momentum in a soccer or football game). The importance of COD speed to match-play has resulted in extensive investigations across various athlete populations as a means of differentiating competition level [1], between positions [2], or to ascertain which technical characteristics are associated with better performance [3]. Nonetheless, to the authors knowledge there has been limited investigation into appropriate means of enhancing COD speed acutely, which could translate to faster performance over the long-term. 

One method which could be used to acutely enhance COD speed is post-activation potentiation (PAP). PAP is the phenomenon that uses the muscle’s contractile history to enhance performance in speed and power activities [4]. Complex training, which typically involves a superset that combines a strength exercise (also referred to as a conditioning activity; CA) immediately followed by a biomechanically similar power-based exercise [5], can be utilized as a potential means of eliciting a PAP response. Current literature is limited on the effect of PAP on COD speed [6,7]. Okuno et al. [6] discovered that repeated-sprint ability (RSA) (6 × 30 m with a COD at 15 m) was improved in badminton athletes after performing half squats at 50–90% one-repetition maximum (1 RM). Importantly, RSA involves a large proportion of linear sprinting, and consequently may not be an accurate representation of a PAP effect on COD speed [8]. Therefore, there is a need to determine whether undertaking a PAP protocol which utilizes an appropriate CA is a viable means of eliciting acute improvements in COD speed.

Tobin and Delahunt [9] suggested that the stimulus provided by plyometric exercises may cause a similar degree of potentiation typically provided by a strength exercise. In support of this, Tobin and Delahunt [9] also revealed that plyometric exercises (e.g., ankle hops, hurdle hops, and drop jumps) were able to enhance vertical jump height (*p* < 0.01) during a rest interval range of 1–5 min. More specific to this study, Turner et al. [10] outlined that at four minutes (min) and eight min post-CA of alternate leg bounds, 20-m sprint performance was significantly (*p* < 0.001) faster when compared to baseline among strength-trained individuals. A plyometric exercise could have value to potentiating COD speed due to relationships between lower-body power and COD speed [11,12,13,14]. Given that biomechanical specificity is important for invoking PAP [4,15], an exercise such as the lateral bound (LB) could have application to COD speed. Furthermore, the LB has been found to relate to COD speed as measured by the 505 COD speed test [12,16]. There may be potential in using the LB to potentiate COD speed measured via a test such as the 505 COD speed test, although this requires further investigation. 

Previous research has also shown that training background can influence whether an individual experiences PAP. Lockie et al. [17] found significant relationships between greater peak power in a 5 repetition-maximum (RM) Bulgarian split-squat with the percent potentiation in 0–5 m, 0–10 m, and 0–20 m sprint intervals 2–8 min post-CA. This may be because stronger, more athletic individuals display elevated myosin light chain phosphorylation and tend to have stronger and larger type II muscle fibers [18]. Potentially, if a plyometric exercise such as the LB is used as a CA, more powerful individuals (i.e., those who can jump further) may be able to experience greater PAP than their lesser counterparts.

Overall, the purpose of this research was to determine whether a plyometric exercise could potentiate COD speed as measured by the 505 COD speed test in college-aged men and women. The chosen CA for this study were LB performed unweighted and weighted with 10% body mass. This followed Turner et al. [10], who used a similar protocol with linear bounds. It was hypothesized that both LB and weighted LB would lead to a faster 505 COD speed performance. It was further hypothesized that weighted LB would induce more of a PAP effect due to the additional overload [10]. Lastly, it was hypothesized that there would be significant relationships between vertical jump height, LB distance and percent potentiation in the 505 COD speed test [17].

## 2. Materials and Methods

### 2.1. Subjects

The study utilised a repeated measures study design consisting of a convenience sample comprised of 40 subjects (23.72 ± 2.45 years; 1.70 ± 0.10 m; 73.33 ± 12.46 kg), including 20 males (24.1 ± 2.71 years; 1.77 ± 0.10 m; 79.08 ± 12.15 kg) and 20 females (23.35 ± 2.08 years; 1.65 ± 0.06 m; and 67.71 ± 9.74 kg). G*Power software (v 3.1.9.3, Universität Kiel, Düsseldorf, Germany) confirmed post hoc that 40 subjects within a 3 × 6 repeated measures analysis of variance (ANOVA) allowed data to be interpreted with an effect level of 0.2, and power level of 0.8, when significance was set at 0.05 [19]. Furthermore, G*Power software confirmed that 40 subjects for the 3 × 2 repeated measures ANOVA ensured data could be interpreted with an effect level of 0.25, and power level of 0.8, when significance was set at 0.05 [19]. Lastly, G*Power software confirmed that for a correlation, point biserial model, a sample size of 40 allowed data to be interpreted with an effect level of 0.4 when the power level was 0.8 and significance was set at 0.05 [19]. Subjects were recruited if they were 18 years of age or older, resistance trained for at least one year, recreationally active at least 2–3 times per week in a field or court sport (i.e., basketball, volleyball, tennis, soccer, lacrosse, flag football, and ultimate frisbee) for at least two years, and free of any lower-extremity injuries for the past year. This study was approved by the university Institutional Review Board (ethics approval number: HSR-18-0002). All subjects received a clear explanation of the study, including risks and benefits of participation. Written informed consent was obtained from subjects prior to testing.

### 2.2. Procedures

Testing was conducted over four days with 48–72 h separating each session which were completed at the same time of day across the sessions for each subject, depending on their availabilities [17,20,21]. The testing protocol involved a jump testing and familiarization session, followed by three sessions completed in a randomized order utilizing three different CAs (LB, weighted LB, and a control condition). Subjects were instructed to refrain from intensive exercise and maintained their typical diet 24 h prior to each testing session. Session one involved the recording of anthropometric data, maximal jump testing, and familiarization of the CA and COD speed test. During session one, the subject’s age, height, and body mass were taken. Height was measured barefoot using a stadiometer (Detecto, Webb City, MO, USA) body mass was measured via an electronic scale (Ohaus, Parisppany, NJ, USA) to the nearest 0.01 kg. This measurement was also used to calculate 10% of the subject’s body mass for the weighted LB intervention. 

A standardized warm-up of 5 min of jogging at a self-selected pace on a treadmill, 10 min of dynamic stretching, and progressive speed runs (~60%, 70%, 80%, and 90% of perceived maximum) over the testing distance was performed at the start of each session. After the warm-up in session one, subjects performed three vertical jump and LB from each leg to indirectly measure vertical and lateral lower-body power. Session Two through Four consisted of the same standardized dynamic warm-up before the subject performed two trials of the 505 COD speed test (one per leg) at 5 and 2.5 min prior to the CA. The order of trials for each leg was reliant on the subject’s dominant leg, which was defined as the subject’s preferred cutting leg [22]. The order of the legs tested during the 505 COD speed test was kept consistent for each subject for all trials. After baseline measurements, one of the three CAs (LB, weighted LB, or control condition) was selected at random and completed by the subject, before they completed the 505 COD speed test at ~15 s, 4, 8, 12, and 16 min post-CA [10,17,20,21,23,24]. Testing sessions were all conducted on an indoor, wooden sprung basketball court. Subjects were free to wear their own footwear, and the same footwear was worn for all testing sessions.

### 2.3. Vertical Jump

The vertical jump is arguably the most common test used to indirectly measure lower-body power in the vertical plane [25], and thus was included in this study. A Vertec apparatus (EPIC Athletic Performance, Lincoln, NE, USA) was used to measure vertical jump performance. This equipment has been extensively utilized in jump performance testing, and established procedures adopted within previous studies as well as the applied setting were undertaken [2,16,25,26,27,28,29,30,31,32,33,34,35,36]. The procedures for determining jump height demonstrated acceptable levels of reliability (intra-class correlation coefficient (ICC) = 0.91; typical error of measurement (TEM) = 4.36; and coefficient of variation (CV) = 7.86%). The subject stood side-on to the Vertec with their dominant side. Keeping their heels on the floor, the subject fully extended their arm to displace as many vanes as possible, the last vane removed was used as the zero reference. Without a preparatory step, the subject jumped and tapped the highest vane possible. Jump height was measured in centimeters (cm). No restrictions on knee angle or countermovement were placed on the subject. Each subject performed three trials with 2 min of recovery between each trial, with the mean of the trials used for analysis.

### 2.4. Lateral Bound (LB)

As LB were the CA in this study, they were also used to indirectly measure single-leg lateral power via established procedures [12,16,37]. Both the left (ICC = 0.97; TEM = 0.04; CV = 2.86%) and right (ICC = 0.94; TEM = 0.06; CV = 4.04%) lateral bounds demonstrated acceptable levels of reliability. The use of LB as a measure of single-leg lateral power has previously been used within the scientific literature is common practice within the applied setting [12,16,37], due to the limited equipment required for testing and the ease of conducting testing procedures. The testing procedures require the subject to stand on their testing leg with the medial aspect of their foot level with the start line. Subjects are able to use a self-selected range of motion during the movement. The subject jumped laterally as far as possible, landing on two feet. Distance was recorded from the start line to the lateral margin of the testing leg with measuring tape (Lufkin, Apex Tool Group, Sparks, MD, USA) to the nearest 0.01 m. If the subject did not “stick” the landing, the trial was disregarded and reattempted. Three trials were performed with 2 min rest between each trial. The mean lateral jump for each leg distance was used for analysis. The dominant leg was determined by the leg with the greater LB distance [38,39].

### 2.5. 505 COD Speed Test

**Figure 1:**
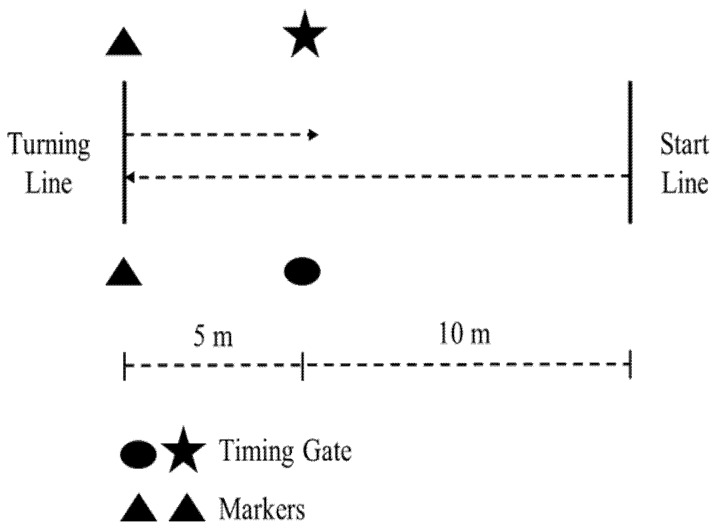
Structure of the 505 change-of-direction (COD) speed test.

The 505 COD speed test, shown in Figure 1, involves a 180° turn in between two 5-m sprints. Methodology for the 505 COD speed test was performed per previous methods [12,13,16,37,40,41,42,43]. The subject began at the start line and accelerated through the photocell timing gate (Brower Timing Systems, Draper, UT, USA) to the turning line indicated by a line marked on the basketball court. The subject then placed either their left or right foot, depending on the trial, on or behind the turning line, performed a 180° direction change before sprinting back through the gate. If the subject changed direction before the turning line or with the incorrect foot, the trial was disregarded and reattempted. Time was recorded to the nearest 0.01 seconds (s) by the photocell timing gates, and the mean of the trials completed at each time point was used for analysis. The only exception was for the baseline values, which was the mean of the four trials from 5 min and 2.5 min pre-CA) [10,17,20,21,23,24]. As noted, the dominant leg was tested first at each time interval, and was defined as the subject’s preferred cutting leg [22].

### 2.6. PAP Interventions

As stated, following the dynamic warm-up, subjects performed two trials of the 505 COD speed test (one per leg, with the order kept consistent across all time points) at 5 and 2.5 min prior to the CA with the mean of all CA calculated to provide baseline data [10,17,20,21,23,24]. The reliability of baseline testing for the 505 COD speed test, demonstrated acceptable levels (ICC = 0.98; TEM = 0.04; CV = 1.54%). After recording the baseline data, subjects performed one of three CAs. One session involved unweighted LB as the CA, where subjects had to perform 3 sets of 5 LB per leg (30 bounds in total) [10]. Another session involved 3 sets of 5 weighted LB per leg (30 bounds in total), with a resistance of 10% body mass provided by a weighed vest [10]. For both LB conditions, subjects were instructed to stand on their right leg and jump laterally to the left as far as possible and had to “stick the landing” on their left leg. Once on their left leg, subjects jumped laterally to the right as far as possible and landed on their right leg and had to “stick the landing”. This sequence was performed continuously until 5 bounds were completed for each leg. The work:rest ratio for this exercise was 1:1. For example, if a subject took 10 s to complete the set, they would rest for 10 s before starting the next set of LBs. For the control condition, subjects were seated for 4 min, which was the approximate duration for the LB CAs [17,20,21]. After the CA, subjects completed two trials of the 505 COD speed test at each of the following time points: ~15 s, 4, 8, 12, and 16 min post-CA [10,17,20,21,23,24]. The subjects were not informed as to what their preceding 505 COD speed test times were to eliminate the influence of feedback [17]. The mean of the two trials performed at each time point was used for analysis. 

Post-CA COD performance percentage change was calculated by using the following equation: % Potentiation = Potentiated Variable (COD at ~15 s, 4, 8, 12, and 16 min) ÷ Unpotentiated Variable (average baseline) × 100. Any value that was equal to 100 indicated that no potentiation had occurred; any value that was less than 100 indicated that positive potentiation had occurred (i.e., faster 505 COD speed test performance); and any value that was greater than 100 concluded that negative potentiation had occurred (i.e., slower 505 COD speed test performance) [17,20,21,44].

### 2.7. Statistical Analysis

The Statistics Package for Social Sciences (Version 24.0; IBM Corporation, New York, NY, USA) was used to compute all analyses. Descriptive statistics (mean ± standard deviation (SD)) were calculated for all subjects. Normality of the data was assessed by visual analysis of the Q-Q plots [45,46]. For the relative reliability analysis, ICC were used to determine trial-to-trial variability of assessed variables. An ICC ≥ 0.70 was considered acceptable [47,48]. Absolute reliability of discrete measures was assessed by TEM [49,50,51]. The TEM was calculated through the formula: TEM = Standard Deviation × √(1 − ICC). The CV was expressed as a percentage, which was calculated by the formula CV = 100 × ((1 – ((test score – TEM) ÷ test score)) [49,52]. A CV of less than 10% was set as the criterion for reliability [53,54]. 

The sexes were combined in this analysis as both men and women can experience potentiation [55], and this approach has also been used in other studies [21,56]. A 3 × 6 repeated measures ANOVA (condition (LB, weighted LB, and control condition) × time (baseline, 15 s, 4, 8, 12, and 16 min)) was used to measure within-subject 505 COD speed test performance across the different time points. Similar analyses have been conducted in previous studies [10,17,21,57]. Best potentiated performance was also analyzed to view individual responses for each subject regardless of the time point which it occurred [17,21,58]. A 3 (LB, weighted LB, and control condition) × 2 (baseline and best 505 COD speed test) repeated measures ANOVA was used for this analysis. If a significant F ratio was detected in any ANOVA calculations, post hoc pairwise comparisons were conducted using the Bonferroni adjustment procedure for multiple comparisons. Furthermore, the smallest worthwhile change (SWC) was also calculated to measure the within-subject 505 COD speed test performance across the different time points, as well as best potentiated performance regardless of the time point at which it occurred. The SWC was determined by multiplying the between-subject standard deviation by 0.2, which is the typical small effect [59,60]. 

To investigate relationships between lower-body power measured by the vertical jump and LB with percent 505 COD speed test potentiation, partial correlations were used. Partial correlations controlling for sex was selected due to the established differences between men and women in power and speed tests [26,37]. Similar to the explanation provided by Dillman Carpentier and Stevens [61], sex was coded in the statistical analysis (males = 1; females = 2) such that the analysis could be conducted. Furthermore, numerous published studies in high-impact journals have used partial correlations to control for the confounding effects of sex so as to investigate relationships between different physical fitness [28,37,62,63] and other scientific variables [61,64,65]. The correlation strength was designated as: an r between 0 to ±0.3 was small; ±0.31 to ±0.49, moderate; ±0.5 to ±0.69, large; ±0.7 to ±0.89, very large; and ±0.9 to ±1 near perfect for relationship prediction [66]. For all analyses, significance was set at *p* < 0.05.

## 3. Results

All variables were deemed to be normally distributed as determined by the Q–Q plot analysis. Descriptive data for the 505 COD speed test following the LB, weighted LB, and control condition are shown in Table 1, while the percent potentiation is displayed in Table 2. When considering the 505 COD speed test data recorded from across all time points post-CA relative to baseline, there was no significant main effect for condition (F_2,38_ = 0.175, *p* = 0.840, partial η^2^ = 0.004) or condition x time (F_10,30_ = 0.453, *p* = 0.919, partial η^2^ = 0.011). There was a significant main effect for time (F_5,35_ = 2.666, *p* = 0.023, partial η^2^ = 0.196). However, post hoc analyses indicated that there were no significant pairwise differences between 505 COD speed test time across the time points (*p* = 0.131–1.000). With regards to the best potentiated 505 COD speed test time, there was again no significant main effect for condition (F_2,38_ = 0.133, *p* = 0.875, partial η^2^ = 0.003) or condition x time (F_2,38_ = 0.736, *p* = 0.482, partial η^2^ = 0.019). There was a significant main effect for time (F_1,39_ = 106.717, *p* < 0.001, partial η^2^ = 0.732). The pairwise comparison showed that the best potentiated 505 COD speed test time was significantly faster than the baseline (*p* < 0.001).

The SWC provides a statistically derived value for determining whether a player has made a ‘real’ significant improvement in performance beyond their random variations in time that will be produced with multiple trials. The SWC to indicate a ‘real’ improvement in performance were 0.06 s when performing the 505 COD speed test. These SWC values exceeded that of the TEM (0.04 s) for the 505 COD speed test calculated within this study. The results of the SWC aligned with that of the repeated measures ANOVA as no meaningful change in 505 COD speed test performance is evident for condition or condition x time. The best potentiated 505 COD speed test time did produce an effect greater than the SWC across all conditions. 

The mean vertical jump for the subjects in this study was 53.84 ± 13.35 cm (males = 63.18 ± 11.50 cm; females = 44.51 ± 7.08 cm). For the dominant leg, the mean LB was 1.44 ± 0.24 m (males = 1.59 ± 0.23 m; females = 1.30 ± 0.14 m); the non-dominant leg LB mean was 1.37 ± 0.23 m (males = 1.51 ± 0.22 m; females = 1.24 ± 0.15 m). Table 3 displays the correlation data between vertical jump and LB performance and the percent potentiation for LB, weighted LB, and control condition. There were no significant correlations between the lower-body power measures and 505 COD speed test percent potentiation for any of the CA. 

## 4. Discussion

The purpose of this study was to determine if LB and weighted LB could potentiate COD performance as measured by the 505 COD speed test in college-aged men and women. The results did not support the hypothesis that LB performed both unweighted and weighted with 10% body mass could acutely improve the 505 COD speed test. Even though the best potentiated 505 COD speed test performance following the LB or weighted LB was faster, they were no different to the 4-min rest control condition. Further, there were no significant relationships between jump performance (vertical jump and LB from the dominant and non-dominant legs) with percent potentiation. These results may have occurred because the LB did not provide enough of an overload stimulus, which is generally needed to invoke PAP [4,55]. However, what should be noted is that although the LB and weighted LB did not provide the necessary stimulus needed to invoke PAP in the 505 COD speed test, it did not hinder COD speed, and with an individualized recovery period, could potentiate this quality. This could have practical application for strength and conditioning coaches who could combine plyometrics and COD drills, knowing that with appropriate recovery COD speed could be maintained.

There were no significant or meaningful changes to 505 COD speed test times for the college-aged men and women at any of the measured time points following the LB or weighted LB. The lack of a significant change in 505 COD speed time was despite the biomechanical specificity between the LB and 180° cut featured in the 505 COD speed test. A factor which has previously been associated with inducing potentiation as measured via performance [12,16]. Perhaps, the LB, even when weighted, may not provide the necessary overload required to invoke PAP [4,55]. This could be influenced by the unique requirements of changing direction. Spiteri et al. [3] noted that faster female basketball players exerted 30 newtons per kg body mass during the braking phase in the 180° cut from the 505 COD speed test. This provides an example of the high forces needed to change direction. Potentially, greater stress is needed from a CA in order to potentiate a COD action. Future research could investigate heavy resistance exercises such as a back or front squat [67], or the barbell hip thrust [68,69], to identify whether those types of exercises can acutely improve the 505 COD speed test.

The results indicated that when scaled to the best potentiated performance (i.e., the fastest 505 COD speed test time regardless of time), the 505 COD speed test time was faster than that of baseline. This suggests that both the LB and weighted LB could acutely enhance the 505 COD speed test; however, any improvements were not different to the control condition of 4 min rest. Practically, this finding indicates that a 4 min rest period may be just as effective at improving 505 COD speed test times as a weighted lateral bound or lateral bound conditioning activity for some individuals. The variability between individuals within the current study and how they may demonstrate a potentiated performance at different time points, and to a different degree following the conditioning activities is similar to previous research [7,58,68], and highlights the great variability between individuals and how they will respond to conditioning activities. Overall, despite the results not appearing to be positive (i.e., the subjects experienced potentiated 505 COD speed test times where they performed bounds or just rested), what should be noted is that the 505 COD speed test did not appear to be negatively affected by the prior performance of plyometrics. This is notable for strength and conditioning practitioners. Training programs targeting COD speed often incorporate a range of exercises, including plyometrics and COD speed drills [27,70]. Plyometric exercises such as the LB or weighted LB could be performed prior to drills that feature actions similar to the 505 COD speed test. As long as appropriate rest intervals are implemented, COD speed should not be hindered, and may in some instances be potentiated. This could lead to strength and conditioning coaches efficiently structuring their training sessions to optimize athlete development and improvement in COD speed. 

The partial correlations revealed no significant relationships between lower-body power in the vertical or horizontal plane and 505 COD speed test percent potentiation. This is in contrast to previous research which has suggested that stronger individuals, and those with superior training history, could experience greater PAP [17,18,44,55]. However, the subjects in the current study were recreationally trained, and their vertical jump and LB performance were lesser than that from higher level athletes. For example, the males and females in this study had a mean vertical jump of 63.18 ± 11.50 cm and 44.51 ± 7.08 cm, respectively. Junior college football players had a mean vertical jump of ~0.70 cm [2], while Division I football players had a mean vertical jump of ~0.80 cm [1]. Considering both legs, the mean LB from this study was approximately ~1.40 m. Following specific speed and agility training, recreationally-trained males and females had a LB of ~1.80 m [27]. Potentially, relationships between vertical jump and LB with PAP could be different in higher level or more trained athletes; this requires further investigation. Nonetheless, the data from this study indicated no relationship between greater lower-body power and PAP in the 505 COD speed test performed by college-aged men and women.

There are several limitations to this study that should be acknowledged. The subject population were recreational athletes. Given that higher-level athletes may respond differently to a CA [44], the results from this study may not apply to more elite populations. Only two plyometric exercises were analyzed in this study (LB and weighted LB). Potentially, other plyometric exercises could potentiate the 505 COD speed test, such as ankle hops, hurdle hops, and drop jumps [9]. Only one type of COD speed test was analyzed in this study. There are a wide range of COD tests available to practitioners, which have different movement demands [8,71]. The observational methodology and T-patterns present within the study may have also been of influence, although the procedures utilized are highly prevalent within the scientific literature [10,11,16]. Future research should investigate whether other movements common to COD speed tests (e.g., cuts made at different angles to the 180° COD in this study) can be potentiated by a specific CA.

## 5. Conclusions

The current study revealed that 3 × 5 LB or 3 × 5 weighted LB (additional load equal to 10% body mass) were not able to enhance COD speed as measured by the 505 COD speed test college-aged, recreationally-trained men and women. By looking at the best individual results, LB and weighted LB were able to acutely improve 505 COD speed test time, but no more than a control condition of 4 min rest. This indicates that for some individuals, a conditioning activity of 4 min passive rest is just as effective as undertaking weight LBs or LBs in inducing a potentiated state. Further, there were no significant relationships between lower-body power measured by the vertical jump and LB with 505 COD speed test percent potentiation. These data suggest relatively limited impact of plyometrics on COD performance measured by the 505 COD speed test. Although the LB did not provide the stimulus to cause an acute improvement in 505 COD speed test performance, it did not appear to hinder COD speed. As both LB and weighted LB did not negatively affect 505 COD speed test time, strength and conditioning coaches could incorporate plyometrics prior to COD drills to enhance training efficiency. There should be no detriment (with potential enhancement) to COD speed as long as appropriate and individualized recovery periods are utilized.

## Figures and Tables

**Table 1:** Descriptive statistics (mean ± SD) for the 505 COD speed test following the PAP interventions (lateral bounds (LB), weighted lateral bounds (WLB), and control condition (CC)).

Data Collection Time Points	LB (s)	WLB (s)	CC (s)
Baseline	2.77 ± 0.27	2.77 ± 0.28	2.78 ± 0.30
15 s~	2.77 ± 0.29	2.75 ± 0.26	2.75 ± 0.33
4 min	2.73 ± 0.30	2.72 ± 0.27	2.73 ± 0.32
8 min	2.75 ± 0.31	2.75 ± 0.29	2.73 ± 0.33
12 min	2.74 ± 0.29	2.74 ± 0.28	2.73 ± 0.33
16 min	2.73 ± 0.27	2.74 ± 0.30	2.73 ± 0.31
Best	2.65 ± 0.26	2.66 ± 0.26	2.65 ± 0.31

**Table 2:** Percent potentiation (%) compared to baseline across all time points post-lateral bounds (LB), weighted lateral bounds (WLB), and control condition (CC).

Data Collection Time Points	LB	WLB	CC
15 s~	99.79 ± 4.47	99.29 ± 4.15	100.12 ± 4.98
4 min	98.53 ± 4.04	98.46 ± 4.44	99.24 ± 3.84
8 min	98.98 ± 5.05	99.54 ± 5.20	99.09 ± 3.55
12 min	98.85 ± 4.33	98.14 ± 5.09	99.38 ± 4.13
16 min	98.32 ± 4.02	99.14 ± 5.02	99.08 ± 3.77
Best	95.75 ± 3.99 *	96.03 ± 3.49 *	96.58 ± 3.05 *

* Significantly (*p* < 0.05) different from baseline.

**Table 3:** Correlation data between the vertical jump (VJ), and dominant and non-dominant leg LB, and percent potentiation in the 505 COD speed test following the PAP conditions (lateral bound (LB), weighted lateral bound (WLB), or control condition (CC)).

Condition	VJ		Dominant Leg LB		Non-Dominant Leg LB	
	*r*	*p*	*r*	*p*	*r*	*p*
LB						
15 s	0.103	0.532	−0.136	0.410	−0.181	0.271
4 min	0.054	0.745	−0.017	0.920	−0.019	0.909
8 min	0.202	0.218	0.195	0.235	0.132	0.424
12 min	0.150	0.363	−0.215	0.188	−0.237	0.147
16 min	−0.018	0.913	−0.184	0.263	−0.195	0.234
WLB						
15 s	0.013	0.935	0.178	0.277	0.210	0.200
4 min	0.027	0.872	0.062	0.709	0.073	0.661
8 min	0.129	0.435	−0.024	0.887	0.014	0.932
12 min	0.099	0.548	0.155	0.346	0.150	0.362
16 min	0.045	0.785	−0.045	0.787	−0.031	0.853
CC						
15 s	−0.158	0.336	−0.283	0.081	−0.292	0.071
4 min	−0.052	0.754	−0.124	0.452	−0.090	0.585
8 min	0.031	0.852	0.077	0.641	0.001	0.993
12 min	0.021	0.897	−0.167	0.310	−0.161	0.328
16 min	−0.201	0.220	−0.301	0.063	−0.308	0.056

## Abbreviations

The following abbreviations are used in this manuscript:

COD	Change-of-direction
PAP	Post-activation potentiation
CA	Conditioning activity
*p*	Significance
m	Meter
RSA	Repeated-sprint ability
RM	Repetition-maximum
Min	Minute
LB	Lateral bound
r	Correlation coefficient
VJ	Vertical jump
WLB	Weighted lateral bound
kg	Kilograms
ANOVA	Analysis of variance
CC	Control condition
cm	Centimeters
s	Seconds
SD	Standard deviation
